# The importance of molecular weight in determining the minimum dose of oat β-glucan required to reduce the glycaemic response in healthy subjects without diabetes: a systematic review and meta-regression analysis

**DOI:** 10.1038/s41430-022-01176-5

**Published:** 2022-06-29

**Authors:** Jarvis C. Noronha, Andreea Zurbau, Thomas M. S. Wolever

**Affiliations:** 1 INQUIS Clinical Research Ltd. (formerly GI Labs), Toronto, ON Canada; 2 Toronto 3D Knowledge Synthesis and Clinical Trials Unit, Clinical Nutrition and Risk Factor Modification Centre, St. Michael’s Hospital, Toronto, ON Canada; 3 Department of Nutritional Sciences, Temerty Faculty of Medicine, University of Toronto, Toronto, ON Canada; 4 Present Address: School of Medicine, Faculty of Medicine, The University of Queensland, Brisbane, QLD Australia

**Keywords:** Nutrition, Metabolism

## Abstract

To determine the minimum amount of oat β-glucan (OBG) required to reduce glycaemic responses (MinDose), we conducted a systematic review and meta-regression analysis of acute, crossover, single-meal feeding trials that examined the effects of adding OBG or oat bran to a carbohydrate-containing test-meal versus a control test-meal containing an equivalent amount of available-carbohydrate (avCHO) from the same or similar source. Medline, Embase, and Cochrane Library were searched up to 18 August 2021. The primary outcome was glucose incremental-area-under-the-curve (iAUC). Secondary outcomes included insulin iAUC, and glucose and insulin incremental peak-rise (iPeak). Two independent reviewers extracted data. Results were expressed as ratio-of-means (RoM) with 95% confidence intervals (CIs). Linear associations were assessed by random effects meta-regression. MinDose was defined as the dose at which the upper 95% CI of the regression line cut the line of no effect (i.e., RoM = 1). Fifty-nine comparisons (*n* = 340) were included; 57 in healthy subjects without diabetes and two in subjects with diabetes; 24 high-MW (>1000 kg/mol), 22 medium-MW (300–1,000 kg/mol), and 13 low-MW (<300 kg/mol). In healthy subjects without diabetes the associations between OBG dose and glucose iAUC and iPeak were linear (non-linear *p* value >0.05). MinDoses for glucose iAUC for high-MW, medium-MW and low-MW OBG, respectively, were estimated to be 0.2 g, 2.2 g and 3.2 g per 30 g avCHO; MinDoses for glucose iPeak were less than those for iAUC. Insufficient data were available to assess MinDose for insulin, however, there was no evidence of a disproportionate increase in insulin. More high-quality trials are needed to establish MinDose in individuals with diabetes.

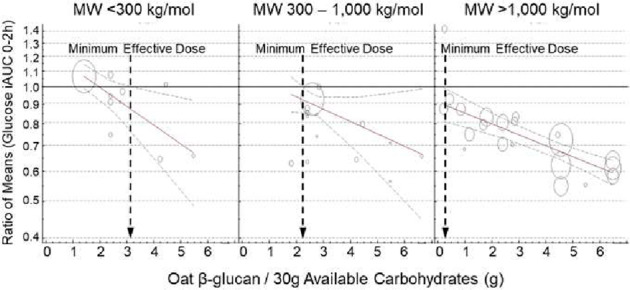

## Introduction

β-glucan, a viscous soluble dietary fibre found naturally in oats and barley, has several potentially beneficial physiological effects including reducing postprandial glycaemic responses (PPGR) [1, 2] and serum cholesterol [3–5]. In 2010, the ability of β-glucan to reduce PPGR was established in a scientific opinion by a European Food Safety Authority (EFSA) Panel review of six human intervention studies [6–11] that concluded that 4 g of either oat β-glucan (OBG) or barley β-glucan (BBG) per 30 g available carbohydrates (avCHO) is required to obtain a consistent reduction in PPGR [12]. However, these findings were limited due to the small number of studies available and the assumption that OBG and BBG have similar effects on PPGR even though they differ with respect to the ratio of β-(1 → 3) to β-(1 → 4) linkages, molecular weight (MW), solubility and conformation [13]. Additionally, the EFSA review did not take into consideration potentially important confounding variables such as, β-glucan MW, nature of the comparator, health status, food form, study methodology quality, duration of follow-up, and risk of bias in their conclusions.

These limitations were addressed in a recent systematic review and meta-analysis (SRMA) that investigated the effect of OBG on PPGR and postprandial insulinemic responses (PPIR) [2]. A total of 35 reports containing data for 103 trial comparisons involving 538 participants were identified and included in the meta-analysis. There was high certainty in the evidence for the reduction of PPGR and PPIR by OBG (median dose, 2.8 g per 30 g avCHO; range, 0.1–22.6 g per 30 g avCHO), however, OBG dose, OBG MW and the type of comparator were identified as significant effect modifiers [2]. The results of this SRMA [2] indicated a linear relationship between OBG and PPGR, and that OBG could reduce PPGR at lower doses than the 4 g OBG per 30 g avCHO required by EFSA to make a health claim related to OBG and PPGR. However, the minimum dose of OBG required to lower PPGR was not explored. Therefore, we aimed to conduct a systematic review and meta-regression analysis (SRMRA) by dose of randomized and non-randomized acute, crossover, single-meal, controlled feeding trials to identify the minimum dose of OBG that needs to be added to a test meal to reduce PPGR.

## Methods

This SRMRA was conducted according to the Cochrane Handbook for Systematic Reviews of Interventions [14]. Data were reported in accordance with the Preferred Reporting Items for Systematic Reviews and Meta-Analyses (PRISMA) guidelines [15]. The study protocol is an extension of a previous SRMA [2] which was registered on the Open Science Forum (OSF) registry [16].

### Data sources

MEDLINE, EMBASE, and the Cochrane Central Register of Controlled Trials were searched through August 18, 2021. Electronic searches were supplemented with manual searches of references from included studies. The detailed search strategy is outlined in Supplementary Table 1.

### Study selection

We included randomized and non-randomized acute, crossover, single-meal, controlled feeding trials that examined the effects of adding OBG or oat bran (high in OBG) to a carbohydrate-containing meal in humans regardless of age, sex, or health condition. For a trial comparison to be included, the comparator (control) test meal had to contain an equivalent amount of avCHO from the same or similar source as the OBG test-meals (matched control, e.g., OBG-containing spaghetti vs. OBG-free spaghetti). To minimize bias and heterogeneity trial comparisons where the comparator (control) test-meals had different sources of avCHO (unmatched control, e.g., OBG muffins vs. glucose, or OBG-containing spaghetti vs. white bread) were excluded. Protein, fat and glycaemic index (GI) have independent effects on glucose and insulin responses [17]. Thus, differences in protein, fat and GI between the OBG and control test-meals would be expected to confound the effect of OBG. Indeed, the nature of the comparator (e.g., matched and unmatched) was found to be a significant effect modifier in the reduction of PPGR by OBG in our previous SRMA [2]. We also excluded trials of parallel design, chronic feeding, studies in which participants were not fasting at baseline, and trials that did not provide appropriate outcome data, did not report OBG MW, used non-oat sources of β-glucan (e.g., barley β-glucan, BBG), and used oats or oat flour as the source of OBG.

We excluded parallel studies to avoid the bias and heterogeneity arising from the fact that they compare the PPGR after the test food in one group of subjects to that after the control food in a different group of subjects. The mean glucose iAUC elicited by 50 g oral glucose 2 groups of different healthy subjects without diabetes of similar age, sex and BMI can differ, in one study with *n* = 8–10 by as much as ±28% of the mean [18] and in another with *n* = 13–15 by ±13% of the mean [19]. We excluded BBG because its effect on PPGR may differ from that of OBG due to its different molecular structure [13]. We excluded oats and oat flour as sources of OBG because they contain ~13 g avCHO per gram of OBG along with bioactive compounds such as protein, fat, flavonoids (e.g., anthocyanins) [20] and phenolics (e.g., avenanthramides) [21]. These confound the effect of OBG because the glycaemic impact of oats not only can differ by as much as 30–40% depending on the method of processing [22] but also may differ from that of the control test-meal.

### Data extraction

Two investigators (JCN and AZ) independently reviewed and extracted relevant data from each included report. Extracted data included participant characteristics (e.g., health status, age, sex, BMI), OBG dose (expressed as g OBG/30 g avCHO [12]), OBG MW (<300 kg/mol = low; 300 to ≤1000 kg/mol = medium; >1000 kg/mol = high), intervention and comparator meal characteristics (nature of foods [e.g., glucose, bread, muffins, pasta, juice] and macronutrient composition [total carbohydrate, total fibre, soluble fibre, avCHO, protein, fat]), study design, duration of follow-up, setting, funding sources, and outcome data. In the absence of numerical values for outcome data and the inability to reach study authors, values were extracted from figures using Plot Digitizer, version 2.5.1 (Free Software Foundation, Boston, MA). If outcome data were provided for multiple follow-up durations (e.g., 120 min and 180 min) in a single study, the data points for the 120 min were used to minimize heterogeneity.

Native OBG has a MW of 2000–2200 kg/mol. We divided MW into categories of low, medium and high, with cut-points of <300, 300–1000 and >1000 kg/mol because of evidence that OBG with MW > 1000 would have a similar effect on glycemic response as native OBG, whereas once MW had been reduced to <300 kg/mol., the effect would be significantly smaller. Specifically, when compared to a muffin with no OBG, muffins containing 4 g OBG with MWs of 130, 380, 590 and 2190 kg/mol reduced glucose iAUC by 2, 10, 27 and 23%, respectively, and muffins containing 8 g OBG with MWs of 220, 410, 760 and 2230 kg/ml reduced glucose iAUC by 31, 29, 45 and 46% respectively [23].

### Outcomes

The primary outcome was glucose incremental area under the curve, ignoring area below the baseline (iAUC). Secondary outcomes included glucose iPeak and insulin iAUC and iPeak.

### Data synthesis and analysis

Data were expressed as a ratio of means (RoM) with 95% confidence intervals (CIs). RoM is a method to present continuous measures on a ratio scale and is calculated by dividing the mean value in the intervention group by the mean value in the control group (Supplementary Fig 1). This method facilitates clinical interpretation (e.g., RoM of 1.2 indicates an increase of 20% in the intervention group compared to the control group; a RoM of 0.7 indicates a reduction of 30%) and controls for baseline differences in the comparator groups across studies [24–26]. Paired analyses were applied to all comparisons [26, 27]. If multiple comparisons were available in the same population, we controlled for unit of analysis error by dividing the N of the respective arm by the number of times it was included.

All analyses were completed using STATA software version 16.1 (StataCorp, College Station, TX, USA). Meta-regression analyses were conducted by dose (net OBG content per 30 g avCHO between test and control) for each MW category (low, medium, and high) as MW was shown to be a significant effect modifier in the reduction of PPGR by OBG in our previous SRMA [2]. Within each MW category, meta-regression analyses were also separated by health status and follow-up duration (i.e., trials of 120 mins follow-up duration in participants without type 2 diabetes (T2D) and trials of 120–180 mins follow-up duration in participants with and without T2D). Meta-regression analyses were only conducted if there were six or more trial comparisons available for each MW category and sub-category of health status and follow-up duration [28]. Linear associations were assessed by random effects meta-regression and non-linear thresholds were assessed with restricted cubic splines with three knots at Harrell’s recommended centiles (10%, 50%, 90%) [29].

The minimum dose of OBG added to a test meal that elicits a statistically significant reduction in postprandial glycaemic response (MinDose) was taken to be the dose at which the upper 95% CI of the regression line cut the line of no effect (i.e., RoM = 1).

## Results

### Search results

Supplementary Fig 2 shows the literature search and selection process. Of 1669 reports identified, 1497 were excluded based on titles and abstracts. Of 172 reports reviewed in full, 152 were excluded based on eligibility criteria. A total of 20 reports containing data for 59 trial comparisons in 340 participants were included in the final analyses [7, 8, 23, 30–46].

### Trial characteristics

**Fig. 1:**
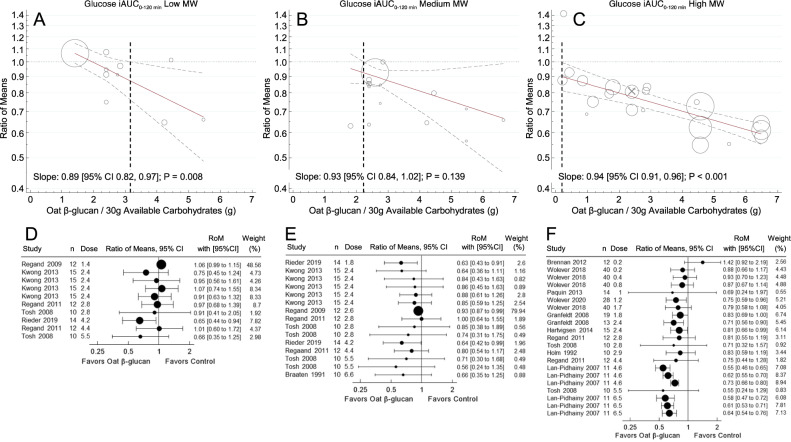
Meta-regression analysis of trials with 120 min follow-up durations assessing the effect of OBG dose on glucose iAUC in subjects without diabetes. Footnotes MW, molecular weight; OBG, oat β-glucan; iAUC, incremental area under the curve; avCHO, available carbohydrate; RoM, ratio of means; CIs, confidence intervals. **A**–**C:** Pooled dose-response relationships for: (**A**) low MW (<300 kg/mol); (**B**) medium MW (300–1000 kg/mol); and (**C**) high MW (>1,000 kg/mol) OBG on glucose iAUC. Individual comparisons are represented by the circles, with the weight of each result in the overall analysis represented by the size of the circles: open circles, healthy subjects; circle containing an “×”, subjects with metabolic syndrome. Solid lines are the estimated linear dose responses; gray dashed lines, upper and lower 95% CIs; vertical black dashed lines, minimum OBG dose required to reduce glucose iAUC with 95% certainty. **D-F**: effect of OBG on glucose iAUC (expressed as RoM with 95% CI) for individual studies with: (**D**) low; (**E**) medium; and (**F**) high MW OBG. Trial comparisons are sorted from the lowest (top) to the highest (bottom) dose of OBG per 30 g avCHO.

Supplementary Table 2 shows the individual characteristics of all included trials and trial comparisons. The follow-up duration of most trial comparisons was 120 min (76%), with the rest being 180 min (24%). All trials were in outpatient settings and conducted in North America (75%) and Europe (25%). Trial funding came from agency sources (51%), industry sources (27%), or both (10%), with no funding information reported in 12% of the trials. Participants were males and females (52% male, 48% female) aged (median (range) of the reported means) 37 (24, 63) years with a BMI of 24.8 (22.4, 31.1) kg/m². Nearly all the available comparisons were conducted in healthy participants (95%) with 2 (3%) in individuals with T2D (3%), and 1 (2%) in individuals with metabolic syndrome (MetS). Results for subjects with T2D were excluded from the analysis of “healthy subjects”. However, the results for subjects with MetS were included with those of “healthy subjects” because the identification of MetS requires measurement of blood pressure, glucose, HDL, triglycerides and waist circumference, all 5 of which are not usually measured in acute glycemic response studies. Thus, a group of “healthy” subjects with no history of diabetes probably includes several who have MetS. Furthermore, the definition of MetS has varied with time and in different guidelines, and the results of the 1 study in people with MetS [34] are indistinguishable from those in the other subjects (Fig. 1F).

The interventions involved consumption of OBG added to various foods including glucose/dextrose solutions (27%), muffins (24%), porridges/oatmeal (17%), muesli (14%), bread (10%), snack bars/products (3%), pasta (3%), and juices (2%), and the same foods without OBG. A median (range) of 4.4 (0.2, 11.7) g OBG was added to a median 50 (25, 64) g avCHO, for a median OBG dose per 30 g avCHO of 3.2 (0.3, 7.0) g.

### MinDose for glucose iAUC

Figure 1 shows the meta-regression analysis of trials with 120 min follow-up duration assessing the effect of OBG on glucose iAUC. In individuals without T2D (i.e., healthy and MetS) the MinDoses were 3.2 g for low-MW OBG, 2.2 g for medium-MW OBG and 0.2 g for high-MW OBG per 30 g avCHO. Dose-response trends were linear except for in the medium-MW category which suggested a non-linear relationship (*p* = 0.036) between glucose iAUC and dose. Removal of Regand 2009 [40] resulted in the trend being linear (non-linear *p* value = 0.189).

When trials with 120–180 min follow-up duration assessing the effect of OBG on glucose iAUC in individuals with and without T2D were included, the results were similar with MinDoses of 3.1 g for low-MW OBG, 2.2 g of medium MW-OBG and 0.2 g of high MW-OBG per 30 g avCHO (Supplementary Fig 3).

The MinDoses associated with 10, 15 and 20% reductions in glucose iAUC (the dose at which the upper 95% CI of the regression line cuts RoM = 0.9, 0.85 and 0.8), respectively, are 1.4 g, 2.1 g and 2.8 g per 30 g avCHO for high-MW OBG but could not be determined for med- and low-MW OBG because the upper 95% CI was ROM ≥ 0.9. The lowest doses (g/30 g avCHO) used in any single study that elicited a statistically significant reduction in glucose iAUC (RoM [95%CI]) were: 4.2 g (0.65 [0.44–0.94]) for low-MW, 1.8 g (0.63 [0.43–0.91]) for medium-MW, and 1.2 g (0.75 [0.59–0.96]) for high-MW (Fig. 3A, Supplementary Table 3).

### MinDose for glucose iPeak

**Fig. 2:**
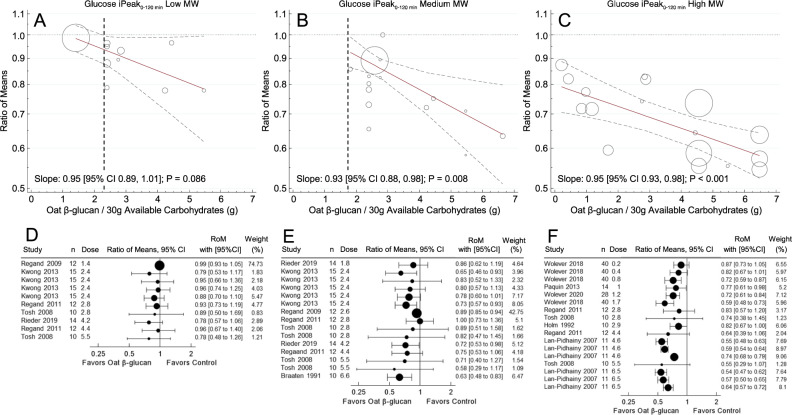
Meta-regression analysis of trials with 120 min follow-up durations assessing the effect of OBG dose on glucose iPeak in subjects without diabetes. Footnotes MW, molecular weight; OBG, Oat β-glucan; iPeak, peak increment; avCHO, available carbohydrate; RoM, ratio of means; CIs, confidence intervals. **A**–**C:** Pooled dose-response relationships for: (**A**) low MW (<300 kg/mol); (**B**) medium MW (300–1000 kg/mol); and **C**, high MW (>1000 kg/mol) OBG on glucose iPeak. Individual comparisons are represented by the circles, with the weight of the study in the overall analysis represented by the size of the circles: open circles, healthy subjects. Solid lines are the estimated linear dose responses; gray dashed lines, upper and lower 95% CI is; vertical black dashed lines, minimum OBG dose required to reduce glucose iPeak with 95% certainty (there is no such line on panel C because the upper 95% CI is <y = 1 at the lowest dose studied, 0.2 g/30 g avCHO). **D**–**F**: effect of OBG on glucose iAUC (expressed as RoM with 95% CI) for individual studies with: (**D**) low; (**E**) medium; and (**F**) high MW OBG. Trial comparisons are sorted from the lowest (top) to the highest (bottom) dose of OBG per 30 g avCHO.

Figure 2 shows the meta-regression analysis of trials with 120 min follow-up duration assessing the effect of OBG on glucose iPeak. In individuals without T2D (i.e., healthy and MetS), the MinDoses were 2.3 g for low-MW OBG, 1.8 g for medium-MW OBG and <0.2 g of high-MW OBG per 30 g avCHO. Dose-response trends for all MW categories were linear (non-linear *p* value > 0.05).

When trials with 120–180 min follow-up duration assessing the effect of OBG on glucose iPeak in individuals with and without T2D were included, the MinDose for low MW-OBG could not be determined because the upper 95% CI of the regression line was >1 across the entire dose range studied, but the MinDoses for medium-MW (1.6 g) and low-MW (<0.2 g) were similar to those for 120 min studies in subjects without diabetes (Supplementary Fig 4).

**Fig. 3:**
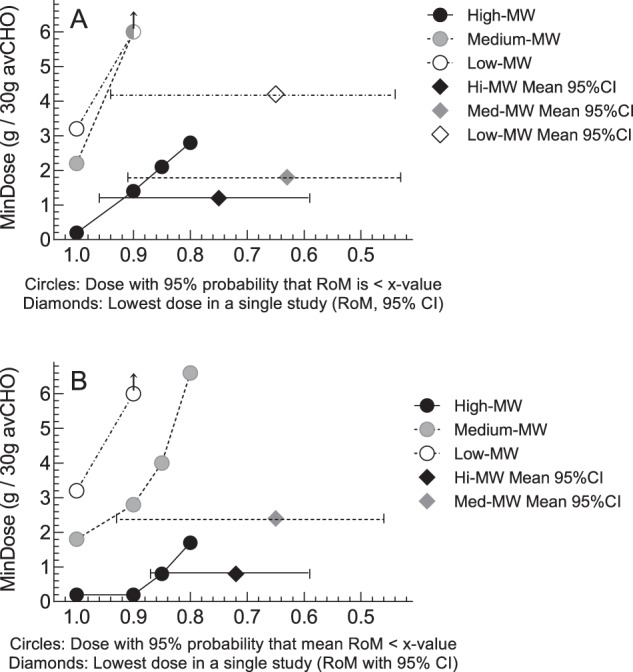
Minimum OBG dose required to lower glucose iAUC and glucose iPeak by MW in participants without type 2 diabetes. Footnotes Panel (**A**) minimum dose of oat β-glucan (OBG; g per 30 g available carbohydrate), required to reduce glucose iAUC (MinDose). Panel (**B**): MinDose of OBG to reduce glucose iPeak. Both panels: circles show the MinDoses (y-axis) associated with a 95% probability that RoM is <*x*-axis values of 1.0, 0.9, 0.85 and 0.8 (i.e., mean reductions of >0%, >10%, >15% and >20%, respectively). Black, gray and open circles, respectively, are for high-MW (>1000 kg/mol) medium-MW (300–1000 kg/mol) and low-MW (<300 kg/mol) OBG. Arrows on circles indicate that MinDose is > the largest dose studied. Black, gray and open diamonds, respectively, show the dose and RoM ± 95% confidence intervals of the lowest dose of high-MW, medium-MW and low-MW OBG in any single study to elicit a statistically significant reduction in glucose iAUC (Panel **A**) or glucose iPeak (Panel **B**). There is no open diamond in Panel B because no study elicited a significant reduction in glucose iPeak.

The MinDoses associated with 10, 15 and 20% reductions in glucose iPeak, respectively (the dose at which the upper 95% CI of the regression line cuts RoM = 0.9, 0.85 and 0.8), were 2.8 g, 4.0 and 6.6 g per 30 g avCHO for medium-MW OBG and <0.2 g, 0.8 g and 1.7 g per 30 g avCHO for high-MW OBG, but could not be determined for low-MW OBG because the upper 95% CI was ROM ≥ 0.9. The lowest dose (g/30 g avCHO) used in any single study that elicited a statistically significant reduction in glucose iPeak (RoM [95% CI]) was: 2.4 g (0.65 [0.46–0.93]) for medium-MW, and 0.8 g (0.72 [0.59–0.87]) for high-MW; none of the low-MW studies showed a statistically significant reduction in glucose iPeak (Fig. 3B, Supplementary Table 3).

### MinDose for insulin iAUC and iPeak

Due to insufficient data (<6 trial comparisons for each MW / health status/study duration), the MinDose for insulin iAUC and iPeak could not be determined. However, nine publications reported data for 16 comparisons of insulin iAUC (Supplementary Table 4) and eight comparisons of iPeak outcomes (Supplementary Table 5) [7, 8, 31, 33–35, 39, 44, 46]. The RoM was not significantly <1 for either glucose or insulin RoM for 16 comparisons, <1 for glucose but not insulin for four comparisons, <1 for insulin but not glucose for 1 comparison, and <1 for both glucose and insulin for three comparisons (Supplementary Tables 4 and 5). The RoMs for glucose and insulin did not differ significantly for any of the 24 comparisons (Supplementary Tables 4 and 5, Supplementary Fig. 5). The RoMs (mean ± SEM) for glucose iAUC and insulin iAUC for the *n* = 14 comparisons in subjects without diabetes, 0.79 ± 0.025 and 0.81 ± 0.034, respectively, were both less than 1 (*P* < 0.001) and did not differ from each other (*P* = 0.55). Similarly, mean ± SEM RoM for glucose iPeak and insulin iPeak, respectively, for the *n* = 8 comparisons in subjects without diabetes, 0.79 ± 0.045 and 0.75 ± 0.052, respectively, were similar to those for iAUC, were both less than 1 (*P* = 0.002) and did not differ from each other (*P* = 0.32).

## Discussion

The results of this SRMRA showed that, in healthy subjects without diabetes, the minimum doses of OBG added to a food or test meal that are associated with a reduction in mean glucose iAUC over 120 min with 95% certainty (MinDose) were 0.2 g per 30 g avCHO for high-MW OBG, 2.2 g per 30 g avCHO for medium-MW OBG and 3.2 g for low-MW OBG per 30 g avCHO. The respective MinDoses for glucose iPeak were <0.2 g, 1.8 g and 2.3 g per 30 g avCHO. Not enough data were available to determine MinDose for insulin iAUC and iPeak. However, in the available trials, reductions in glucose iAUC or iPeak occurred without a disproportional increase in insulin iAUC or iPeak.

In the context of our analysis, MinDose means that doses of OBG ≥ MinDose will be associated with a RoM <1 with 95% certainty, or, in other words, that the food with OBG will elicit a lower mean glucose iAUC or iPeak than the food without OBG with ≥95% certainty. This does not necessarily mean that the difference in glucose response will be either statistically or physiologically significant. This approach could be justified because, to make a claim, EFSA did not indicate the measure of glycaemic response to be used, the effect-size required nor whether the difference had to be statistically significant [12]. Nevertheless, we also provide MinDose values for various effect sizes and the need for statistical significance.

The ability to demonstrate a statistically significant difference in a cross-over trial depends on the number of subjects and the magnitude of their within-individual variation. Within-individual coefficient of variation (CV_within_) of glucose iAUC varies widely in different laboratories. In a study involving 28 labs around the world, lab mean CV_within_ varied from 12 to 40% [47]. With CV_within_ of 12–40%, respectively, *n* = 10 provides 80% power detect significant differences of 17% or 56%; if *n* = 90, differences of 5% or 17% can be detected. The factors that determine the magnitude of CV_within_ are not well understood. An early study suggested that CV_within_ of glucose iAUC is highest in type 1 diabetes, intermediate in subjects without diabetes and lowest in subjects with T2D [48]. Also, it has been suggested that CV_within_ is: high with avCHO intakes <20 g [49]; lower with capillary versus venous blood sampling [18, 50]; reduced by consuming coffee or tea with the test meal versus water [51]; high when glucose is measured using a method with low analytical precision [52, 53]; and not reduced by controlling subject fasting time, providing a standard dinner and prohibiting vigorous exercise the previous day [54]. However, the effect of these procedures on CV_within_ is not always seen [47].

The ability to demonstrate a statistically significant reduction of PPGR also depends on the % reduction targeted. It is not clear how big a difference in PPGR is physiologically relevant. Health Canada opined that the minimum physiologically relevant difference in glucose iAUC is 20% [55]. However, smaller differences may be important. For example, a 6-month randomized controlled trial of 210 subjects with T2D and baseline HbA1c of 7.1% showed that a 14% reduction in diet glycaemic index reduced HbA1c by 0.32% relative to control (*p* < 0.001) [56], an effect size that both the US Food and Drug Administration [57] and the European Medicines Agency have considered to be clinically meaningful [58]. Thus, in addition to showing the minimum OBG doses associated with a mean RoM<1 with 95% certainty, we also provide dose thresholds associated with a mean RoM <0.9, <0.85 and <0.8 (reductions of 10%, 15% and 20%, respectively), and indicate the lowest dose used in any single study showing a statistically significant reduction in PPGR (Fig. 3, Supplementary Table 3).

Our previous SRMA [2] included 103 trial comparisons and found that OBG reduced glucose iAUC and iPeak by 23% and 28% and insulin by 22% and 24%, respectively. Dose, MW and comparator were identified as significant effect modifiers, and significant linear dose-response relationships were observed for all four outcomes. The current analysis differs in two ways. Previously we examined the dose-response relationship by pooling the slopes from the trial comparisons, a method that forces the regression line through (0,1) and results in 95% CIs which are <RoM = 1. Here we performed meta-regression of the effect sizes by dose which allows the 95% CIs of the regression line to be >RoM = 1 and, thus, suggests that MinDose is the point where the upper 95% CIs of the regression line cuts a specified y-value. Also, since the previous study identified the nature of the comparator as a significant effect modifier, we only included matched comparisons to minimize bias (e.g., effect of avCHO source).

The mechanism by which viscous fibres such as OBG reduce PPGR is thought to be related to their ability to increase the viscosity of the contents of the gastrointestinal tract (GIT) as has been demonstrated in in-vitro [59–61], animal [62] and human [41, 60, 63] studies. Viscosity, in turn, is controlled by the concentration and MW of the OBG [60]. The present results highlight the importance of OBG MW in that, although the slopes of the regression lines of RoM on dose are similar for low-, medium- and high-MW OBG, the reduction in PPGR at any dose of OBG is greatest for high-MW, and lowest for low-MW OBG (Figs. 1 and 2). Thus, MinDose critically depends on MW, with the MinDose for medium-MW and low-MW OBG, respectively, being more than 10 and 15 times greater than that for high-MW OBG. The MW of OBG incorporated into foods may be reduced by food processes involving high temperature or pressure, such as baking or extrusion [64], but these effects can be controlled [65]. Furthermore, organic acids, such as those present in fruits, have been shown to reduce the MW of OBG [66], particularly in pasteurized drinks [67]. The latter is relevant because we included Paquin et al. 2013 [39] who reported the effect of high MW OBG in a pasteurized mixture of pineapple, orange and banana juices. However, it seems unlikely that the OBG used in the Paquin et al. study was severely degraded since the RoM for glucose iAUC, 0.69, was smaller than that of other studies using a similar OBG dose, 0.75–0.87 (Fig. 1F). The smaller RoM is likely because the authors reported netAUC [68] in which area below fasting is subtracted from area above fasting. Estimating iAUC (ignores area below fasting) from the glucose response curves yields a RoM of 0.80, which is consistent with other studies using a similar OBG dose. The large 95%CI in the Paquin et al. study could be due, at least in part, to the method of AUC calculation and to infrequent blood sampling (0, 30, 60, 90 and 120 min), methodological features both of which tend to increase variation [68].

Nevertheless, the effect of OBG on PPGR also depends on its solubility, or bioavailability, which, in turn, may vary depending on the food matrix (e.g., solution, porridge, bread, cookies etc.) [40, 41] and food storage conditions [38]. Furthermore, the viscosity of glucose solutions containing OBG is not always related to their glycaemic impact [37] since the concentration, and hence viscosity, of OBG solutions within the stomach may be reduced by gastric fluid secretions [69]. High viscosity could reduce glycaemic responses by delaying gastric emptying or reducing the rate of digestion and absorption of carbohydrates in the small intestine, or both; however, the exact mechanism is not completely understood and needs to be investigated further.

Our analysis has several limitations. Nearly all the trial comparisons (95%) were in subjects without diabetes. There were two trials in subjects with T2D. Although our findings did not change materially with the addition of these trial comparisons, we cannot conclude with a high degree of certainty that the MinDoses estimated in subjects without diabetes would apply to those with diabetes. In addition, there were insufficient data to conduct reliable meta-regression analyses for insulin outcomes. However, the results demonstrate that reductions in PPIR occurred in parallel to those for PPGR and, therefore, indicate there is no disproportionate increase in insulin. Other potential limitations were the inclusion of non-randomized studies and the need for using Plot Digitizer. However, of the 20 studies included, there was concern about the randomization process for 1 study [46] and Plot Digitizer was only needed for one study [30]. Neither of these studies were included in the main results (Figs. 1 and 2) because the study follow-ups were >180 min, nevertheless, their results were consistent with those of the other studies (Supplementary Figs. 3 and 4). Our literature search went up to 18 August 2021. A PubMed search on 31 May 2022 (the date of provisional acceptance) from 18 August 2021 with “beta-glucan” in the title returned 153 hits, none of which were eligible for inclusion.

Finally, the primary method we used to determine the MinDose, i.e., the smallest amount that elicits a mean reduction >0% (RoM < 1) with 95% certainty, may not be considered appropriate by other scientists or regulatory authorities. For this reason, we provided results for some other methods of determining MinDose. What is very clear from these results, however, is that whatever method is used to establish MinDose, the amount is far less than the 4 g/30 g avCHO currently required by EFSA [12]. Furthermore, if a food product containing OBG is to be allowed to make a claim for reduced PPGR without having to provide data showing that the product reduces PPGR, then MW of the OBG in the product must be known.

We conclude that the estimated minimum amounts of OBG per 30 g avCHO required to reduce mean glucose iAUC (or iPeak) in healthy subjects without diabetes with 95% certainty are: 0.2 g (<0.2 g) for high-MW OBG, 2.2 g (1.8 g) for medium-MW OBG and 3.2 g (2.3 g) for low-MW OBG. For high-MW OBG, 1.4 g (<0.2 g), 2.1 g (0.8 g) and 2.8 g (1.7 g) per 30 g avCHO, respectively, are required to obtain at least 10%, 15% or 20% reductions in iAUC (iPeak). More high-quality trials are needed to determine the MinDose for insulin responses and MinDose for glucose and insulin responses in subjects with T2D.

## Supplementary information

Supplemental Material

## Data Availability

The datasets generated during and/or analysed during the current study are available from the corresponding author on reasonable request.
