# Supplementary material for: The importance of molecular weight in determining the minimum dose of oat β-glucan required to reduce the glycaemic response in healthy subjects without diabetes: a systematic review and meta-regression analysis
Source: Eur J Clin Nutr. 2022 Jun 29;77(3):308–15. doi: 10.1038/s41430-022-01176-5 (PMC10017511; doi:10.1038/s41430-022-01176-5)
Supplement: Supplementary file 1 — Supplemental Material [file 41430_2022_1176_MOESM1_ESM.docx]

Table of Contents

[Supplementary Table 1. Search strategy 2](#_Toc99699159)

[Supplementary Table 2. Detailed characteristics of included studies 3](#_Toc99699160)

[Supplementary Table 3. Summary of meta-regression findings from trials of 120min follow-up duration assessing the minimum OBG dose required to lower glucose iAUC and iPeak by MW in participants without type 2 diabetes 6](#_Toc99699161)

[Supplementary Table 4. Comparison of glucose and insulin ratio of means (RoM) for incremental area under the curve. 7](#_Toc99699162)

[Supplementary Table 5. Comparison of glucose and insulin ratio of means (RoM) for incremental peak rise. 8](#_Toc99699163)

[Supplementary Figure 1. Ratio of Means (RoM) formulas 9](#_Toc99699164)

[Supplementary Figure 2. Summary of literature search and selection 10](#_Toc99699165)

[Supplementary Figure 3: Meta-regression analysis of trials with 120-180min follow-up durations assessing the effect of OBG on glucose iAUC. 11](#_Toc99699166)

[Supplementary Figure 4: Meta-regression analysis of trials with 120-180 min follow-up durations assessing the effect of OBG on glucose iPeak. 12](#_Toc99699167)

[Supplementary Figure 5. Insulin responses vs glucose responses 13](#_Toc99699168)

[Supplementary References 14](#_Toc99699169)

# **Supplementary Table 1.** Search strategy

| **MEDLINE**  **1946 to Aug 18, 2021** | **EMBASE**  **1946 to Aug 18, 2021** | **Cochrane Central Register of Controlled Trials**  **Through Aug 18, 2021** |
| --- | --- | --- |
|  |  |  |
| 1. oat.tw. | 1. oat.tw. | 1. Avena.ti,ab,kw. |
| 2. Oats.tw. | 2. Oats.tw. | 2. Oat.ti,ab,kw. |
| 3. Avena/ | 3. Avena/ | 3. Oats.ti,ab,kw. |
| 4. Avena*.tw. | 4. Avena*.tw. | 4. Beta-glucans/ |
| 5. beta glucan/ | 5. beta glucan/ | 5. Beta glucan*.ti,ab,kw. |
| 6. beta glucan*.mp. | 6. beta glucan*.mp. | 6. or/1-5 |
| 7. b-glucan*.mp. | 7. b-glucan*.mp. | 7. Glucose/ |
| 8. b-glucan*.mp. | 8. b-glucan*.mp. | 8. glycaemic.mp. |
| 9. or/1-8 | 9. or/1-8 | 9. glycemic.mp. |
| 10. exp Glucose/ | 10. exp Glucose/ | 10. glycemia.mp. |
| 11. glycaemic.mp. | 11. glycaemic.mp. | 11. Insulin/ |
| 12. glycemic.mp. | 12. glycemic.mp. | 12. exp Glucose Tolerance Test/ |
| 13. glycaemia.mp. | 13. glycaemia.mp. | 13. OGTT.mp. |
| 14. glycemia.mp. | 14. glycemia.mp. | 14. or/7-13 |
| 15. exp Glucose Tolerance Test/ | 15. exp Glucose Tolerance Test/ | 15. 6 and 14 |
| 16. OGTT.mp. | 16. OGTT.mp. |  |
| 17. or/10-16 | 17. or/10-16 |  |
| 18. 9 and 17 | 18. 9 and 17 |  |
| 19. limit 18 to animals | 19. limit 18 to animals |  |
| 20. 18 not 19 | 20. 18 not 19 |  |
|  |  |  |

# **Supplementary Table 2.** Detailed characteristics of included studies

| **Study, Year**  **(Supplementary reference)** | **Participants** | **Health Status** | **Age, years** | **BMI, kg/m^2^** | **Intervention** | | | | **Comparator** | | **Duration, mins** | **Setting (OP/IP)** | **Funding** | **Outcome(s)** |
| --- | --- | --- | --- | --- | --- | --- | --- | --- | --- | --- | --- | --- | --- | --- |
|  |  |  |  |  | **OBG Dose, g*** | **avCHO,**  **g†** | **Reported MW,**  **g/mol**‡ | **Description** | **avCHO,**  **g** | **Description** |  |  |  |  |
| Braaten et al. 1991 (1) | 10  (4M, 6F) | Healthy | 25.0+5.8 | 24.9+4.3 | 11.3 | 51.1 | 804,950ƒ | Gel-like pudding containing 50g glucose + 14.5g oat gum | 50 | 50g glucose in 500mL water | 180 | Canada  (OP) | Industry | Glucose iAUC  Glucose iPeak |
| Braaten et al. 1994 (2) | 11  (7M, 4F) | Healthy | 51.9+6.2 | 27.3+2.0 | 8.8 | 60.7 | 900,000¶ | Oat gum + wheat farina | 62.2 | Wheat farina | 180 | Canada  (OP) | Agency & Industry | Glucose iAUC  Glucose iPeak  Insulin iAUC |
|  |  |  |  |  | 8.8 | 56.6 | - | Oat Bran |  |  |  |  |  |  |
|  | 10  (7M, 3F) | T2D | 58.4+5.5 | 27.7+3.0 | 8.8 | 60.7 | 900,000¶ | Oat gum + wheat farina | 62.2 | Wheat farina | 180 |  |  |  |
|  |  |  |  |  | 8.8 | 56.6 | - | Oat Bran |  |  |  |  |  |  |
| Brennan et al. 2012 (3) | 12  (4M, 8F) | Healthy | Range: 18-40 | Range: 22.5-28 | 0.21 | 25 | - | 15% oat bran substitution with wheat flour in ready-to-eat snack product | 25 | Ready-to-eat snack product without oat bran | 120 | UK  (OP) | - | Glucose iAUC |
| Grandfeldt et al. 2008 (4) – series 1 | 19  (6M, 13F) | Healthy | 37.5+15.2 | 22.4+0.6 | 3.0 | 50 | - | Test meal (muesli with flakes made from oat bran (OatWell) + yogurt + sandwich) | 48.5 | Reference meal (yogurt + sandwich) | 120 | Sweden  (OP) | Industry | Glucose iAUC  Insulin iAUC |
| Grandfeldt et al. 2008 (4) – series 2 | 13  (5F, 8M) | Healthy | 37.5+3.6 | 22.4+0.6 | 4.0 | 50 | - | Test meal (muesli with flakes made from oat bran (OatWell) + yogurt + sandwich) | 50 | Reference meal (yogurt + sandwich) |  |  |  |  |
| Hartvigsen et al. 2014 (5) | 15  (7M, 8F) | MetS | 62.8+4.2 | 31.1+3.2 | 4.2 | 50 | 1,978,000 | Wheat bread + concentrated OBG (PromOat) | 50 | Wheat Bread | 120 | Denmark  (OP) | Agency | Glucose iAUC  Insulin iAUC |
| Holm et al. 1992 -AJCN (6) | 10  (3M, 7F) | Healthy | Range:  M, 34-44;  F, 26-49 | Range:  18.5-23.5;  17.5-23.0 | 8.6 | 50 | - | Oat bran bread  (HSFB-ob) | 50 | White wheat bread  (WWB-tl) | 180 | Sweden  (OP) | Industry | Glucose iAUC  Insulin iAUC |
| Holm et al. 1992 -EJCN (7) | 10  (9M, 1F) | Healthy | Range: 23-45 | Range: 18-23.1 | 5.2 | 54.2 | - | Oat bran pasta | 54.2 | Durum pasta | 120 | Switzerland  (OP) | Agency | Glucose iAUC  Glucose iPeak  Insulin iAUC |
| Juntunen et al. 2002 (8) | 20 (10M,10F) | Healthy | 28.5+5.6 | 22.9+2.9 | 5.4 | 50 | 250,000 | OBG-enriched rye bread | 50 | White wheat bread | 180 | Finland  (OP) | Agency & Industry | Glucose iPeak  Insulin iPeak |
| Kwong et al. 2013 – Food Funct (9) | 15  (7M, 8F) | Healthy | 37.2+11.2 | 26.6+4.3 | 4.0 | 50 | 145,000 | 4g Low MW OBG + 50g dextrose Drink (LD) | 50 | 50g dextrose drink without OBG (ND) | 120 | Canada  (OP) | Agency | Glucose iAUC, Glucose iPeak |
|  |  |  |  |  | 4.0 | 50 | 580,000 | 4g High MW OBG + 50g dextrose Drink (HD) |  |  |  |  |  |  |
|  |  |  |  |  | 4.0 | 50 | 145,000 | 4g Low MW OBG + 50g dextrose Gel (4LG) |  |  |  |  |  |  |
|  |  |  |  |  | 4.0 | 50 | 362,500 | 2g High MW OBG + 2g Low MW OBG + 50g dextrose Gel (2H2LG) |  |  |  |  |  |  |
|  |  |  |  |  | 4.0 | 50 | 471,250 | 3g High MW OBG + 1g Low MW OBG + 50g dextrose Gel (3H1LG) |  |  |  |  |  |  |
| Kwong et al. 2013 - BJN (10) | 15  (7M, 8F) | Healthy | 37.2+11.2 | 26.6+4.3 | 4.0 | 50 | 145,000 | 250mL 50g glucose solution with 4g Low MW OBG (250L) | 50 | 250mL 50g glucose solution (250N) | 120 | Canada  (OP) | Agency | Glucose iAUC, Glucose iPeak |
|  |  |  |  |  | 4.0 | 50 | 580,000 | 250mL 50g glucose solution with 4g High MW OBG (250H) |  |  |  |  |  |  |
|  |  |  |  |  | 4.0 | 50 | 145,000 | 600mL 50g glucose solution with 4g Low MW OBG (600L) | 50 | 600mL 50g glucose solution (600N) |  |  |  |  |
|  |  |  |  |  | 4.0 | 50 | 580,000 | 600mL 50g glucose solution with 4g High MW OBG (600H) |  |  |  |  |  |  |
| Lad-Pidhainy et al. 2007 (11) | 11  (4M, 7F) | Healthy | 34.6+10.4 | 24.7+4.8 | 7.6 | 50 | 2,800,000 | Oat bran muffin with 8g OBG  (8-g Fresh) | 50 | Whole Wheat Muffin (Control) | 120 | Canada  (OP) | - | Glucose iAUC, Glucose iPeak |
|  |  |  |  |  | 7.6 | 50 | 2,000,000 | Oat bran muffin with 8g OBG + 2 freeze thaw temperature cycling (8-g 2FT) |  |  |  |  |  |  |
|  |  |  |  |  | 7.6 | 50 | 1,800,000 | Oat bran muffin with 8g OBG + 4 freeze thaw temperature cycling (8-g 4FT) |  |  |  |  |  |  |
|  |  |  |  |  | 10.8 | 50 | 2,700,000 | Oat bran muffin with 12g OBG  (12-g Fresh) |  |  |  |  |  |  |
|  |  |  |  |  | 10.8 | 50 | 2,400,000 | Oat bran muffin with 12g OBG + 2 freeze thaw temperature cycling (12-g 2FT) |  |  |  |  |  |  |
|  |  |  |  |  | 10.8 | 50 | 2,000,000 | Oat bran muffin with 12g OBG + 4 freeze thaw temperature cycling (12-g 4FT) |  |  |  |  |  |  |
| Paquin et al. 2013 (12) | 14  (14M, 0F) | Healthy | 32±9 | 23.9±4 | 1.1 | 35 | - | Fruit juice enriched with OBG (OatWell) | 35 | Fruit Juice without OBG | 120 | Canada  (OP) | Agency & Industry | Glucose iAUC,  Glucose iPeak,  Insulin iAUC  Insulin iPeak |
| Regand et al. 2009 (13) | 12  (6M, 6F) | Healthy | 42.3±14.6 | 28.8±5.5 | 4.0 | 64 | 197,000 | Oat crisp bread | 65 | Wheat crisp bread | 120 | Canada  (OP) | Agency | Glucose iAUC, Glucose iPeak |
|  |  |  |  |  | 4.0 | 42 | 465,000 | Oat pasta | 44 | Whole wheat pasta |  |  |  |  |
| Regand et al. 2011 (14) | 12  (6M, 6F) | Healthy | 27.3±5.5 | 25.6±5.0 | 6.2 | 38 | 57,000 | Oat Granola  (Low MW OBG, 40g avCHO) | 40 | Wheat granola  (40g avCHO) | 120 | Canada  (OP) | Agency | Glucose iAUC, Glucose iPeak |
|  |  |  |  |  | 6.2 | 38 | 435,000 | Oat Granola  (Med MW OBG, 40g avCHO) |  |  |  |  |  |  |
|  |  |  |  |  | 6.2 | 38 | 2,133,000 | Oat Granola  (High MW OBG, 40g avCHO) |  |  |  |  |  |  |
|  |  |  |  |  | 6.3 | 60 | 82,000 | Oat Granola  (Low MW OBG, 60g avCHO) | 58 | Wheat granola  (60g avCHO) |  |  |  |  |
|  |  |  |  |  | 6.3 | 60 | 325,000 | Oat Granola  (Med MW OBG, 60g avCHO) |  |  |  |  |  |  |
|  |  |  |  |  | 6.3 | 60 | 1,996,000 | Oat Granola  (High MW OBG, 60g avCHO) |  |  |  |  |  |  |
| Rieder et al. 2019 (15) | 14 | Healthy | 44.8±13.8 | 24.3±1.7 | 3.8 | 26.2 | 282,000 | Degraded oat bran concentrate bread (Degraded OBCB) | 26 | White wheat bread | 120 | UK  (OP) | Agency | Glucose iAUC, Glucose iPeak |
|  |  |  |  |  | 3.8 | 26.3 | 592,000 | Optimal oat bran concentrate bread (Optimal OBCB) |  |  |  |  |  |  |
|  |  |  |  |  | 1.7 | 26.4 | 421,000 | Low oat bran concentrate bread (Low OBCB) |  |  |  |  |  |  |
| Tosh et al. 2008 (16) | 10  (6M, 4F) | Healthy | 37.6+18.0 | 23.8+4.4 | 4.4 | 47.9 | 130,000 | Oat bran muffins prepared with enzyme to get low MW OBG (4g L) | 51.4 | Whole wheat muffin | 120 | Canada  (OP) | Agency | Glucose iAUC, Glucose iPeak |
|  |  |  |  |  | 4.4 | 47.9 | 380,000 | Oat bran muffins prepared with enzyme to get medium MW OBG (4g M) |  |  |  |  |  |  |
|  |  |  |  |  | 4.4 | 47.9 | 590,000 | Oat bran muffins prepared with enzyme to get high MW OBG (4g H) |  |  |  |  |  |  |
|  |  |  |  |  | 4.4 | 47.9 | 2,190,000 | Oat bran muffins prepared without enzyme (4g N) |  |  |  |  |  |  |
|  |  |  |  |  | 8.2 | 45 | 220,000 | Oat bran muffins prepared with enzyme to get low MW OBG (8g L) |  |  |  |  |  |  |
|  |  |  |  |  | 8.2 | 45 | 410,000 | Oat bran muffins prepared with enzyme to get medium MW OBG (8g M) |  |  |  |  |  |  |
|  |  |  |  |  | 8.2 | 45 | 760,000 | Oat bran muffins prepared with enzyme to get high MW OBG (8g H) |  |  |  |  |  |  |
|  |  |  |  |  | 8.2 | 45 | 2,230,000 | Oat bran muffins prepared without enzyme (8g N) |  |  |  |  |  |  |
| Wolever et al. 2018 (17) | 40  (22M, 18F) | Healthy | 31.5±11.0 | 25.1±3.0 | 1.4 | 27.7 | - | Instant oatmeal + 0.72g oat bran | 27.6 | Instant oatmeal without oat bran | 120 | Canada  (OP) | Industry | Glucose iAUC, Glucose iPeak |
|  |  |  |  |  | 1.6 | 27.7 | - | Instant oatmeal + 1.43g oat bran |  |  |  |  |  |  |
|  |  |  |  |  | 2.0 | 27.9 | - | Instant oatmeal + 2.86g oat bran |  |  |  |  |  |  |
|  |  |  |  |  | 2.8 | 28.1 | - | Instant oatmeal + 5.72g oat bran |  |  |  |  |  |  |
| Wolever et al. 2020 (18) | 28  (16M, 12F) | Healthy | 33.1+10.6 | 24.8+2.3 | 2  (net) | 51.2 | 2,060,000 | Instant oatmeal (27g) + oat bran (10.1g) + standard breakfast (25-27g white bread, 5-7g butter, 11-21g jam, 240mL 2% milk) | 51.3 | Instant oatmeal (27g) + oat bran (3g) + standard breakfast (25-27g white bread, 5-7g butter, 11-21g jam, 240mL 2% milk) | 180 | Canada  (OP) | Industry | Glucose iAUC, Glucose iPeak  Insulin iAUC  Insulin iPeak |
| Wood et al. 1990 (19) | 9  (4M, 5F) | Healthy | 23.6+3.9 | 24.5+4.2 | 11.7 | 50 | 900,000¶ | Glucose drink (50g) + 14.5g oat gum | 50 | Glucose drink (50g) without oat gum | 180 | Canada  (OP) | Industry | Glucose iAUC |
|  | 10  (7M, 3F) | Healthy | 52+7.3 | 27.3+11.7 | 8.9 | 60 | 900,000¶ | Cream of wheat (48g) + 11g oat gum | 60 | Cream of wheat control (68g) without oat gum |  |  |  |  |
| Wood et al. 1994 (20) | 9  (4M, 5F) | Healthy | 31.4+4.9 | 24.8+6.2 | 1.5 | 50 | 804,950ƒ | 50g glucose drink + 1.8g oat gum | 50 | 50g glucose drink without oat gum | 180 | Canada  (OP) | Industry | Glucose iAUC,  Glucose iPeak  Insulin iAUC  Insulin iPeak |
|  |  |  |  |  | 2.9 | 50 | 804,950ƒ | 50g glucose drink + 3.6g oat gum |  |  |  |  |  |  |
|  |  |  |  |  | 5.8 | 50 | 804,950ƒ | 50g glucose drink + 7.2g oat gum |  |  |  |  |  |  |
|  | 11  (6M, 5F) | Healthy | 34.3±10.4 | 24.3+2.5 | 6.4 | 50 | 250,800ƒ | 50g glucose drink + 7.2g oat gum acid-hydrolyzed for 15mins (OG15) | 50 | 50g glucose drink without oat gum |  |  |  |  |
|  |  |  |  |  | 6.4 | 50 | 101,850ƒ | 50g glucose drink + 7.2g oat gum acid-hydrolyzed for 60mins (OG60) |  |  |  |  |  |  |

Footnotes on next page

Footnotes for Supplementary Table 2.

Data represent mean±SD, unless stated otherwise. “-” represents lack of reporting of data. BMI, body mass index; avCHO, available carbohydrate; MW, molecular weight; OP, outpatient; IP, inpatient; M, male; F, female; OW, overweight; OBG, oat β-glucan; iAUC, incremental area-under-the-curve; iPeak, incremental peak-rise; T2D, type 2 diabetes.

* For Holm et al. 1992 – AJCN (6), OBG dose was calculated by taking the difference of the soluble fibre in OSFB-ob and WWB-mg.

‡ Individual study MW categorizations may differ from categories that were used in this systematic review and meta-analysis. In this analysis, low MW OBG were considered to be <300 kg/mol, medium MW were 300 to ≤1,000 kg/mol, and high MW were >1,000 kg/mol.

ƒ Data retrieved from Table 1 of Wood et al. 2000 (21).

¶ Data retrieved from Wood, 2010 (22).

# **Supplementary Table 3.** Summary of meta-regression findings from trials of 120min follow-up duration assessing the minimum OBG dose required to lower glucose iAUC and iPeak by MW in participants without type 2 diabetes

| Method | Result | | Minimum dose of OBG (g/30g avCHO) by MW | | | | | |
| --- | --- | --- | --- | --- | --- | --- | --- | --- |
|  |  |  | To reduce iAUC | | | To reduce iPeak | | |
|  |  |  | Low | Medium | High | Low | Medium | High |
| Upper 95%ile of meta-regression line | 95% probability that mean reduction is greater than: | 0% | 3.2 | 2.2 | 0.2 | 2.3 | 1.8 | <0.2 |
|  |  | 10% | >6 | >7 | 1.4 | >6 | 2.8 | <0.2 |
|  |  | 15% | - | - | 2.1 | - | 4.0 | 0.8 |
|  |  | 20% | - | - | 2.8 | - | 6.6 | 1.7 |
|  |  | |  |  |  |  |  |  |
| Lowest dose with significant reduction in a single study | OBG dose | | 4.2 | 1.8 | 1.2 | na | 2.4 | 0.8 |
|  | RoM [95% CI] | | 0.65  [0.44 to 0.94] | 0.63  [0.43 to 0.91] | 0.75  [0.59 to 0.96] | na | 0.65  [0.46 to 0.93] | 0.72  [0.59 to 0.87] |

OBG = oat β-glucan; MW = molecular weight; iAUC = incremental area under the curve; iPeak = incremental peak rise; RoM = ratio of means; 95% CI = 95% confidence interval.

# **Supplementary Table 4.** Comparison of glucose and insulin ratio of means (RoM) for incremental area under the curve.

| Study | OBG  g/30g avCHO | OBG MW | Duration (min) | Subjects | Glucose iAUC* | | Insulin iAUC* | | Glucose RoM** | Insulin RoM** | P |
| --- | --- | --- | --- | --- | --- | --- | --- | --- | --- | --- | --- |
|  |  |  |  |  | Intervention | Control | Intervention | Control |  |  |  |
| Braaten 1994 (2) | 4.3 | Medium | 180 | Healthy | 110±18 | 151±20 | 5411±40 | 7374±64 | 0.73 [0.48 to 1.11] | 0.73 [0.46 to 1.18] | 0.98 |
|  | 4.3 | Medium | 180 | T2DM | 599±100 | 752±87 | 4297±866 | 5298±1335 | 0.80 [0.53 to 1.20] | 0.81 [0.54 to 1.23] | 0.95 |
|  | 4.7 | High | 180 | Healthy | 108±17 | 151±20 | 5766±632 | 7374±802 | 0.72 [0.48 to 1.07] | 0.78 [0.49 to 1.24] | 0.78 |
|  | 4.7 | High | 180 | T2DM | 602±95 | 752±87 | 5743±861 | 5298±1335 | 0.80 [0.54 to 1.19] | 1.08 [0.74 to 1.60] | 0.31 |
| Granfeldt 2008 (4) | 1.8 | High | 120 | Healthy | 79.7±7.7 | 95.7±8.6 | 21±714 | 22±802 | 0.83 [0.69 to 1.00] | 0.94 [0.77 to 1.13] | 0.39 |
|  | 2.4 | High | 120 | Healthy | 71.2±11.1 | 100.7±13.5 | 11±2 | 19±2 | 0.71 [0.56 to 0.90] | 0.58 [0.49 to 0.69] | 0.21 |
| Hartvigsen 2014 (5) | 2.4 | High | 120 | MetS | 266±27 | 330±36 | 21.2±3.0 | 25.3±3.5 | 0.81 [0.65 to 0.99] | 0.84 [0.64 to 1.10] | 0.83 |
| Holm 1992 AJCN (6) | 1.9 | High | 180 | Healthy | 89.8±13.6 | 98.7±18.6 | 13±1 | 17±2 | 0.91 [0.65 to 1.28] | 0.78 [0.53 to 1.13] | 0.56 |
| Holm 1992 EJCN (7) | 2.9 | High | 120 | Healthy | 64±12.5 | 76.7±12.3 | 15600±3 | 20100±3 | 0.83 [0.59 to 1.19] | 0.78 [0.62 to 0.97] | 0.74 |
| Paquin 2013 (12) | 0.9 | High | 120 | Healthy | 28.6±17.8 | 41.7±13.4 | 6.8±1.6 | 7.3±1.4 | 0.69 [0.24 to 1.97] | 0.93 [0.61 to 1.42] | 0.61 |
| Wolever 2020 (18) | 1.2 | High | 120 | Healthy | 78±10 | 104±13 | 14±1400 | 22±2500 | 0.75 [0.58 to 0.96] | 0.64 [0.51 to 0.81] | 0.38 |
| Wood 1994 (20) | 3.8 | Low | 180 | Healthy | 101±25 | 130±30 | 226±64 | 279±68 | 0.78 [0.40 to 1.51] | 0.81 [0.45 to 1.44] | 0.93 |
|  | 3.8 | Low | 180 | Healthy | 131±25 | 130±30 | 250±43 | 279±62 | 1.01 [0.56 to 1.82] | 0.90 [0.51 to 1.57] | 0.79 |
|  | 0.9 | Medium | 180 | Healthy | 125±24 | 144±22 | 276±44 | 265±62 | 0.87 [0.48 to 1.58] | 1.04 [0.50 to 2.15] | 0.72 |
|  | 1.7 | Medium | 180 | Healthy | 106±14 | 144±22 | 240±45 | 265±64 | 0.74 [0.45 to 1.20] | 0.91 [0.42 to 1.96] | 0.67 |
|  | 3.5 | Medium | 180 | Healthy | 102±20 | 144±22 | 184±50 | 265±64 | 0.71 [0.39 to 1.30] | 0.69 [0.32 to 1.52] | 0.97 |

Characteristics and summary results for glucose and insulin incremental areas under the curve (iAUC) for all studies reporting both measures. OBG = oat β-glucan; MW = molecular weight (low = <300kg/mol, medium = 300-1,000kg/mol, high = >1,000kg/mol); P = significance of difference between glucose RoM and insulin RoM.

* Values are means±SEM.

** Values are ratios of means (RoM) with 95% confidence intervals.

# **Supplementary Table 5.** Comparison of glucose and insulin ratio of means (RoM) for incremental peak rise.

|  | OBG  g/30g avCHO | OBG MW | Duration (min) | Subjects | Glucose iPeak* | | Insulin iPeak* | | Glucose RoM** | Insulin RoM** | P |
| --- | --- | --- | --- | --- | --- | --- | --- | --- | --- | --- | --- |
|  |  |  |  |  | Intervention | Control | Intervention | Control |  |  |  |
| Holm 1992 EJCN (7) | 2.9 | High | 120 | Healthy | 1.8±0.12 | 2.2±0.26 | 307±37 | 390±72 | 0.82 [0.67 to 1.00] | 0.79 [0.57 to 1.08] | 0.85 |
| Paquin 2013 (12) | 0.9 | High | 120 | Healthy | 1.7±0.24 | 2.2±0.16 | 217±41.4 | 241±55.1 | 0.77 [0.61 to 0.98] | 0.90 [0.59 to 1.36] | 0.54 |
| Wolever 2020 (18) | 1.2 | High | 120 | Healthy | 1.6±0.1 | 2.3±0.2 | 250±23 | 426±46 | 0.72 [0.61 to 0.84] | 0.59 [0.48 to 0.72] | 0.14 |
| Wood 1994 (20) | 3.8 | Low | 180 | Healthy | 2.3±0.3 | 2.8±0.4 | 404±86 | 509±97 | 0.82 [0.56 to 1.20] | 0.79 [0.45 to 1.39] | 0.92 |
|  | 3.8 | Low | 180 | Healthy | 2.9±0.3 | 2.8±0.4 | 459±66 | 509±97 | 1.04 [0.73 to 1.48] | 0.90 [0.56 to 1.45] | 0.66 |
|  | 0.9 | Medium | 180 | Healthy | 2.5±0.3 | 3.0±0.3 | 446±76 | 535±148 | 0.83 [0.57 to 1.22] | 0.83 [0.37 to 1.89] | 1.00 |
|  | 1.7 | Medium | 180 | Healthy | 2.1±0.2 | 3.0±0.3 | 338±82 | 535±148 | 0.70 [0.50 to 0.98] | 0.73 [0.31 to 1.70] | 0.94 |
|  | 3.5 | Medium | 180 | Healthy | 1.8±0.2 | 3.0±0.3 | 264±45 | 535±148 | 0.60 [0.42 to 0.86] | 0.49 [0.22 to 1.12] | 0.68 |

Characteristics and summary results for glucose and insulin incremental peak rises (iPeak) for all studies reporting both measures. OBG = oat β-glucan; MW = molecular weight (low = <300kg/mol, medium = 300-1,000kg/mol, high = >1,000kg/mol); P = significance of difference between glucose RoM and insulin RoM.

* Values are means±SEM.

** Values are ratios of means (RoM) with 95% confidence intervals.

**Supplementary Figure 1.** Ratio of Means (RoM) formulas

$$\ln\left( RoM \right)=ln(\frac{mean [intervention]}{mean [control]} )$$

$$SE \left[ \ln\left( RoM \right) \right]=\sqrt{\frac{{SD}^{2} \left[ intervention \right]}{n \left[ pairs \right] \times{mean}^{2} \left[ intervention \right]}+\frac{{SD}^{2} \left[ control \right]}{n \left[ pairs \right] \times{mean}^{2}\left[ control \right]}-\frac{2 \times r \times SD \left[ intervention \right] \times SD \left[ control \right]}{mean \left[ intervention \right] \times mean \left[ control \right] \times n [pairs]}}$$

The natural logarithm transformed ratios were aggregated across studies using the standard generalized inverse variance method. The pooled transformed ratio and pooled SE of the logarithmic-transformed ratio were then back transformed to obtain a pooled ratio and 95% CI (23-25) “r” refers to the correlation between the two measurements and was assumed to be 0.5. “n [pairs]” refers to the number of paired comparisons. If multiple comparisons were present in a study, n [pairs] = (n [total] / # of comparisons).

# **Supplementary Figure 2.** Summary of literature search and selection

**1669**  **Reports identified**

201 🡪 Cochrane Library (1950 to Aug 18, 2021)

984 🡪 EMBASE (1980 to Aug 18, 2021)

476 🡪 MEDLINE (1950 to Aug 18, 2021)

8 🡪 Manual Searches

**1497**  **Excluded based on title/abstract**

453 🡪 Duplicate reports

21 🡪 Conference Abstracts/Papers

147 🡪 Reviews or meta-analyses

4 🡪 Letters

18 🡪 Protocols

11 🡪 Observation/retrospective studies

13 🡪 Case reports

647 🡪 Animal/*in vitro* studies

79 🡪 Unsuitable intervention

26 🡪 Unsuitable endpoints

78 🡪 Long-term (>1day) interventions

**172 Reports reviewed in full**

**152**  **Excluded after full article review**

3 🡪 Duplicate reports

2 🡪 Review, letter

24 🡪 Inadequate comparator

33 🡪 Inadequate intervention period

9 🡪 Insufficient data

1 🡪 Animal/*in vitro* studies

5 🡪 Not English

1 🡪 Observation/retrospective studies

24 🡪 Unsuitable intervention

13 🡪 Unsuitable endpoints

2 🡪 Inadequate study design

2 🡪 Preload study

33 🡪 Oat intervention

**20 Reports included in meta-analysis (n=340)**

Glucose iAUC: 58 trial comparisons (n=320)

Glucose iPeak 52 trial comparisons (n=252)

Insulin iAUC: 16 trial comparisons (n=150)

Insulin iPeak: 8 trial comparisons (n=82)

iAUC, incremental area-under-the-curve; iPeak, incremental peak-rise

# **Supplementary Figure 3**: Meta-regression analysis of trials with 120-180min follow-up durations assessing the effect of OBG on glucose iAUC.


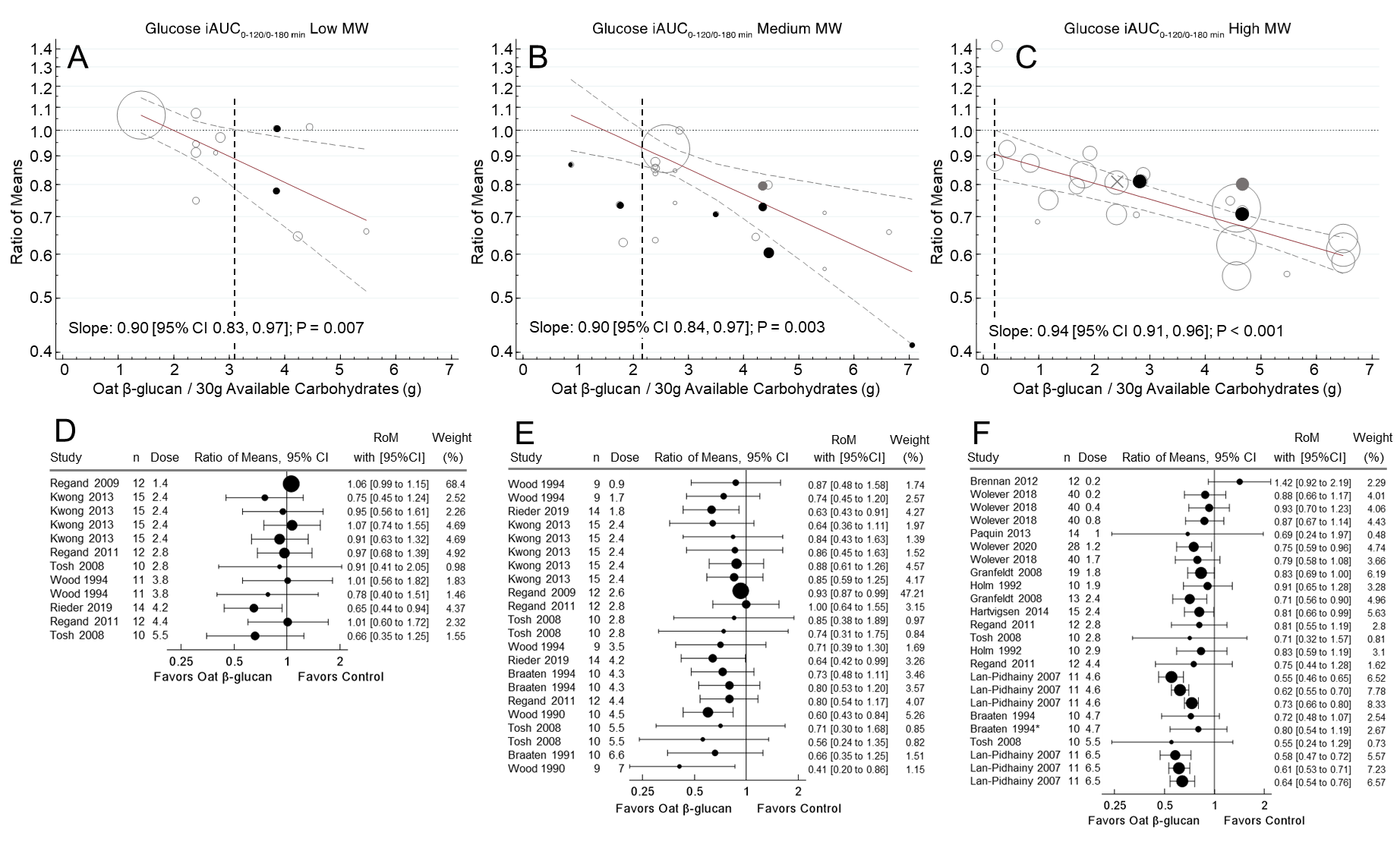


MW, molecular weight; OBG, Oat β-glucan; iAUC, incremental area under the curve; avCHO, available carbohydrate; RoM, ratio of means; CI, confidence intervals. **A-C:** Pooled dose-response relationship for low MW (<300kg/mol) (**A**), medium MW (300-1,000kg/mol) (**B**) and high MW (>1,000kg/mol) (**C**) OBG on glucose iAUC. Individual comparisons are represented by the circles, with the weight of the study in the overall analysis shown by the size of the circles. Open circles = healthy subjects; circle containing an “×” = subjects with metabolic syndrome; filled circles = studies with 180min duration in subjects with (gray) and without (black) type 2 diabetes. Solid lines = the estimated linear dose responses; gray dashed lines = the upper and lower 95% CI; vertical black dashed lines = where the upper 95%CI of the regression cuts y=1 and represents the minimum OBG dose required to reduce glucose iAUC with 95% certainty. **D-F**: effect of OBG on glucose iAUC (expressed as RoM with 95% CI) for individual studies with low (**D**), medium (**E**) and high (**F**) MW OBG. Trial comparisons are sorted from the lowest (top) to the highest (bottom) dose of OBG per 30g avCHO.

# **Supplementary Figure 4**: Meta-regression analysis of trials with 120-180 min follow-up durations assessing the effect of OBG on glucose iPeak.


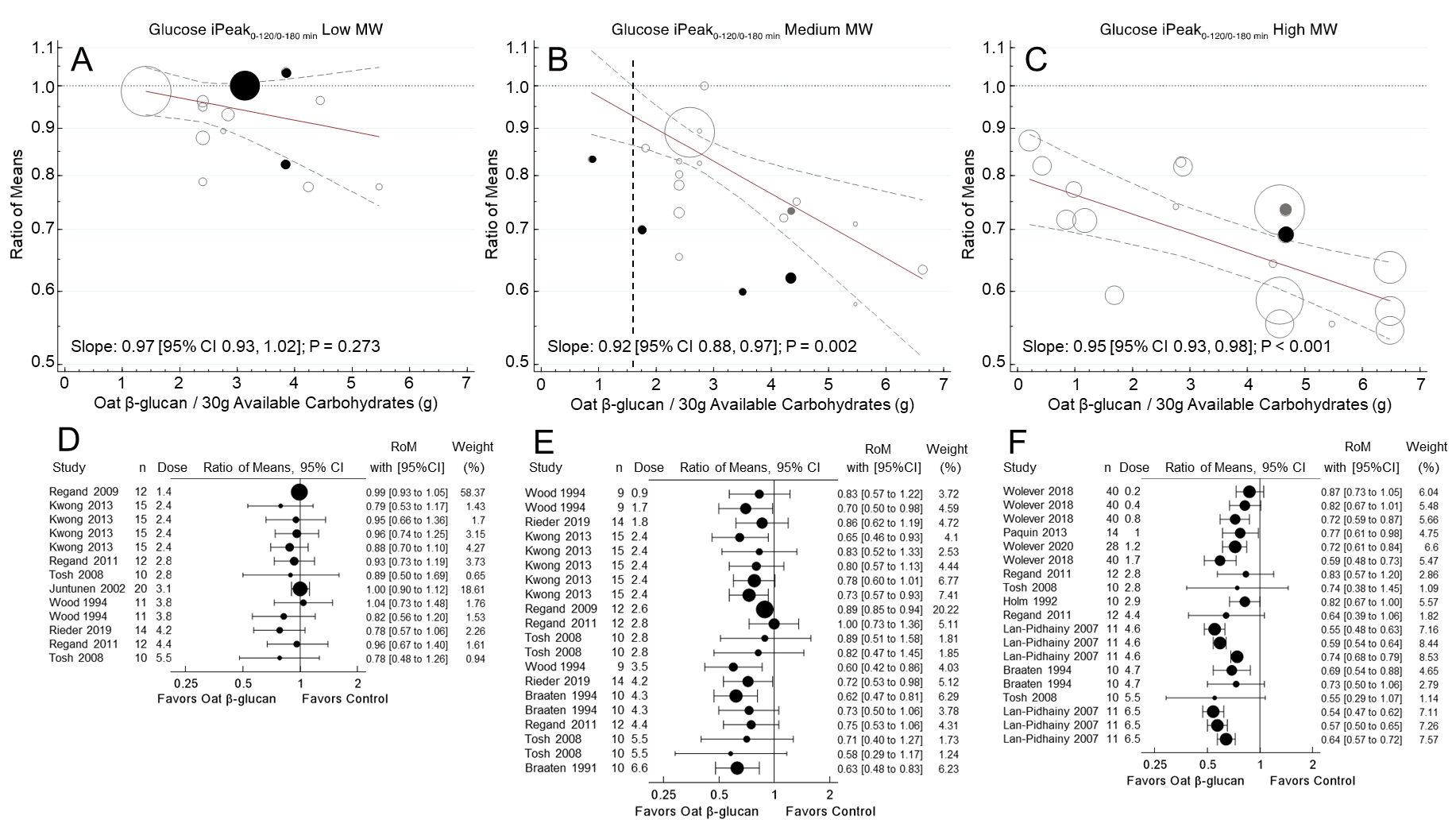


MW, molecular weight; OBG, Oat β-glucan; iPeak, peak increment; avCHO, available carbohydrate; RoM, ratio of means; CI, confidence intervals. **A-C:** Pooled dose-response relationship for low MW (<300kg/mol) (**A**), medium MW (300-1,000kg/mol) (**B**) and high MW (>1,000kg/mol) (**C**) OBG on glucose iPeak. Individual comparisons are represented by the circles, with the weight of the study in the overall analysis represented by the size of the circles. Open circles = healthy subjects; filled circles = studies with 180min duration in subjects with (gray) and without (black) type 2 diabetes. Solid lines = the estimated linear dose responses; gray dashed lines = the upper and lower 95% CI; the vertical black dashed line on panel B = where the upper 95%CI of the regression cuts y=1 and represents the minimum OBG dose required to reduce glucose iPeak with 95% certainty (there is no such line on panel A because the upper 95%CI is > y=1; there is no such line on panel C because the upper 95%CI is < y=1 across the range of doses studied [0.2 – 6.5g/30g avCHO]). **D-F**: effect of OBG on glucose iAUC (expressed as RoM with 95% CI) for individual studies with low (**D**), medium (**E**) and high (**F**) MW OBG. Trial comparisons are sorted from the lowest (top) to the highest (bottom) dose of OBG per 30g avCHO.

# **Supplementary Figure 5.** Insulin responses vs glucose responses

Values are means±SEM for the results shown in Supplementary Tables 4 and 6. Panel A shows ratios of means (RoM) for incremental areas under the curve (iAUC); Panel B shows RoM for incremental peak rises (iPeak). The dashed line is the line of identity.

# **Supplementary References**

1. Braaten JT, Wood PJ, Scott FW, Riedel KD, Poste LM, Collins MW. Oat gum lowers glucose and insulin after an oral glucose load. *Am J Clin Nutr* 1991; **53**: 1425-1430.

2. Braaten, J.T., et al., High beta-glucan oat bran and oat gum reduce postprandial blood glucose and insulin in subjects with and without type 2 diabetes. *Diabet Med*, 1994; **11**: 312-318.

3. Brennan MA, Derbyshire EJ, Brennan CS, Tiwari BK. Impact of dietary fibre-enriched ready-to-eat extruded snacks on the postprandial glycaemic response of non-diabetic patients. *Mol Nutr Food Res* 2012; **56**: 834-837.

4. Granfeldt Y, Nyberg L, Bjorck I. Muesli with 4 g oat beta-glucans lowers glucose and insulin responses after a bread meal in healthy subjects. *Eur J Clin Nutr* 2008; **62**: 600-607.

5. Hartvigsen ML, Gregersen S, Lærke HN, Holst JJ, Bach Knudsen KE, Hermansen K. Effects of concentrated arabinoxylan and beta-glucan compared with refined wheat and whole grain rye on glucose and appetite in subjects with the metabolic syndrome: a randomized study. *Eur J Clin Nutr* 2014; **68**: 84-90.

6. Holm, J. and I. Björck, Bioavailability of starch in various wheat-based bread products: evaluation of metabolic responses in healthy subjects and rate and extent of in vitro starch digestion. *Am J Clin Nutr* 1992; **55**: 420-429.

7. Holm J, Koellreutter B, Wursch P. Influence of sterilization, drying and oat bran enrichment of pasta on glucose and insulin responses in healthy subjects and on the rate and extent of in vitro starch digestion. *Eur J Clin Nutr* 1992; **46**: 629-640.

8. Juntunen KS, Niskanen LK, Liukkonen KH, Poutanen KS, Holst JJ, Mykkanen HM. Postprandial glucose, insulin, and incretin responses to grain products in healthy subjects. *Am J Clin Nutr* 2002; **75**: 254-262.

9. Kwong MG, Wolever TM, Brummer Y, Tosh SM. Attenuation of glycemic responses by oat beta-glucan solutions and viscoelastic gels is dependent on molecular weight distribution. Food Funct 2013; 4: 401-408.

10. Kwong MG, Wolever TM, Brummer Y, Tosh SM. Increasing the viscosity of oat beta-glucan beverages by reducing solution volume does not reduce glycaemic responses. *Br J Nutr* 2013; **110**: 1465-1471.

11. Lan-Pidhainy X, Brummer Y, Tosh SM, Wolever TM, Wood PJ. Reducing Beta-Glucan Solubility in Oat Bran Muffins by Freeze-Thaw Treatment Attenuates Its Hypoglycemic Effect. *Cereal Chem* 2007; **84**: 512-517.

12. Paquin J, Bédard A, Lemieux S, Tajchakavit S, Turgeon SL. Effects of juices enriched with xanthan and beta-glucan on the glycemic response and satiety of healthy men. *Appl Physiol Nutr Metab* 2013; **38**: 410-414.

13. Regand A, Tosh SM, Wolever TM, Wood PJ. Physicochemical properties of beta-glucan in differently processed oat foods influence glycemic response. *J Agric Food Chem* 2009; **57**: 8831-8838.

14. Regand A, Chowdhury Z, Tosh SM, Wolever TMS, Wood P. The molecular weight, solubility and viscosity of oat beta-glucan affect human glycemic response by modifying starch digestibility. *Food Chem* 2011; **129**: 297-304.

15. Rieder A, Knutsen SH, Sainz Fernandez A, Ballance S. At a high dose even partially degraded beta-glucan with decreased solubility significantly reduced the glycaemic response to bread. *Food Funct* 2019; **10**: 1529-1539.

16. Tosh SM, Brummer Y, Wolever TMS, Wood PJ. Glycemic Response to Oat Bran Muffins Treated to Vary Molecular Weight of β-Glucan. *Cereal Chem* 2008; **85**: 211-217.

17. Wolever TMS, Jenkins AL, Prudence K, Johnson J, Duss R, Chu Y. Effect of adding oat bran to instant oatmeal on glycaemic response in humans - a study to establish the minimum effective dose of oat beta-glucan. *Food Funct* 2018; **9**: 1692-1700.

18. Wolever TMS, Tosh SM, Spruill SE, Jenkins AL, Ezatagha A, Duss R, et al. Increasing oat β-glucan viscosity in a breakfast meal slows gastric emptying and reduces glycemic and insulinemic responses but has no effect on appetite, food intake, or plasma ghrelin and PYY responses in healthy humans: a randomized, placebo-controlled, crossover trial. *Am J Clin Nutr* 2020; **11**: 319-328.

19. Wood PJ, Braaten JT, Scott FW, Riedel D, Poste LM. Comparisons of viscous properties of oat and guar gum and the effects of these and oat bran on glycemic index. *J Agric Food Chem* 1990; **38**: 753-757.

20. Wood PJ, Braaten JT, Scott FW, Riedel KD, Wolynetz MS, Collins MW. Effect of dose and modification of viscous properties of oat gum on plasma glucose and insulin following an oral glucose load. *Br J Nutr* 1994; **72**: 731-743.

21. Wood P, Beer M, Butler G. Evaluation of role of concentration and molecular weight of oat β-glucan in determining effect of viscosity on plasma glucose and insulin following an oral glucose load. *Br J Nutr* 2000; **84**: 19-23.

22. Wood PJ. Oat and Rye β‐Glucan: Properties and Function. *Cereal Chem* 2010; **87**: 315-330.

23. Friedrich JO, Adhikari NKJ, Beyene J. The ratio of means method as an alternative to mean differences for analyzing continuous outcome variables in meta-analysis: A simulation study. *BMC Med Res Methodol* 2008; **8**: 32.

24. Friedrich JO, Adhikari NKJ, Beyene J. Ratio of means for analyzing continuous outcomes in meta-analysis performed as well as mean difference methods. *J Clin Epidemiol* 2011; **64**: 556-564.

25. Lajeunesse, MJ. On the meta-analysis of response ratios for studies with correlated and multi-group designs. *Ecology* 2011; **92**: 2049-2055.
